# Public attitudes to Sputnik V vaccination against the novel COVID 19 infection the role of the social-demographic characteristics and pandemic COVID-19 individual experience issues and their implementation as the targets for brief psychosocial intervention

**DOI:** 10.1192/j.eurpsy.2022.1312

**Published:** 2022-09-01

**Authors:** A. Vasileva, N. Neznanov, T. Karavaeva, D. Radionov, A. Yakovlev

**Affiliations:** 1 V. M. Bekhterev National Medical Research Center for Psychiatry and Neurology, Non-psychotic Mental Disorders Treatment And Psychotherapy, saint petersburg, Russian Federation; 2 Bekhterev National Medical Center for Psychiatry and Neurology, Geriatric Psychiatry, Saint-Petersburg, Russian Federation; 3 V. M. Bekhterev National Medical Research Center for Psychiatry and Neurology, Non-psychotic Mental Disorders Treatment And Psychotherapy, saint petersburg, Russian Federation; 4 Saint-Petersburg State University of Aerospace Instrumentation, Statisitcs, saint petersburg, Russian Federation

**Keywords:** covid 19, pandemic, brief psychosocial interevention, vaccine hesitancy

## Abstract

**Introduction:**

Vaccination has proved to be an effective tool in decreasing infectious diseases incidence and their mortality rate. Negative public vaccine attitude can significantly undermine efforts to combat the pandemic that makes vaccine hesitancy one of the WHO main concerns

**Objectives:**

Examination of the relationships in population between vaccine attributes and COVID-19 personal experience, social and demographic characteristics

**Methods:**

Cohort cross-sectional study of the population attitude to vaccination against coronavirus infection COVID-19 was performed online during the first 2 months of mass vaccination in Russia, using the special designed questionnaire assessing social demographic variables, COVID-19 related factors, and preferable sources of information about COVID-19 vaccines. 4977 participants in the age from 18 to 81 years were enrolled in the study to vaccination against coronavirus infection COVID-19 was performed online during the first 2 months of mass vaccination in Russia, using the special designed questionnaire assessing social demographic variables, COVID-19 related factors, and preferable sources of information about COVID-19 vaccines .

**Results:**

34.2% of respondents consider vaccination useful. 31.1% ‑ doubt its effectiveness. 9.9% ‑ consider vaccination unnecessary. 12.2% ‑ dangerous. indifference to vaccination was formed in 7.4% of respondents. They indicated that they do not plan to be vaccinated. 32.3%. postpones their decision until more remote data on the results and effectiveness of vaccination are obtained ‑ 34.0%. were vaccinated at the time of the study ‑ 11.6%.

**Conclusions:**

Attitude towards vaccination depends on age, gender, education, fear of possible complications, coronaphobia. Young people are less focused on vaccination than middle-aged and older people.

**Disclosure:**

No significant relationships.

